# Mass Drug Administration for Trachoma: How Long Is Not Long Enough?

**DOI:** 10.1371/journal.pntd.0003610

**Published:** 2015-03-23

**Authors:** Violeta Jimenez, Huub C. Gelderblom, Rebecca Mann Flueckiger, Paul M. Emerson, Danny Haddad

**Affiliations:** 1 Hubert Department of Global Health, Rollins School of Public Health, Emory University, Atlanta, Georgia, United States of America; 2 International Trachoma Initiative, Task Force for Global Health, Emory University, Atlanta, Georgia, United States of America; 3 Global Ophthalmology Emory, Emory Eye Center, Emory University School of Medicine, Atlanta, Georgia, United States of America; University of California San Francisco, UNITED STATES

## Abstract

**Background:**

Blinding trachoma is targeted for elimination by 2020 using the SAFE strategy (Surgery, Antibiotics, Facial cleanliness, and Environmental improvements). Annual mass drug administration (MDA) with azithromycin is a cornerstone of this strategy. If baseline prevalence of clinical signs of trachomatous inflammation – follicular among 1-9 year-olds (TF1-9) is ≥10% but <30%, the World Health Organization guidelines are for at least 3 annual MDAs; if ≥30%, 5. We assessed the likelihood of achieving the global elimination target of TF1-9 <5% at 3 and 5 year evaluations using program reports.

**Methodology/Principal Findings:**

We used the International Trachoma Initiative’s prevalence and treatment database. Of 283 cross-sectional survey pairs with baseline and follow-up data, MDA was conducted in 170 districts. Linear and logistic regression modeling was applied to these to investigate the effect of MDA on baseline prevalence. Reduction to <5% was less likely, though not impossible, at higher baseline TF1-9 prevalences. Increased number of annual MDAs, as well as no skipped MDAs, were significant predictors of reduced TF1-9 at follow-up. The probability of achieving the <5% target was <50% for areas with ≥30% TF1-9 prevalence at baseline, even with 7 or more continuous annual MDAs.

**Conclusions:**

Number of annual MDAs alone appears insufficient to predict program progress; more information on the effects of baseline prevalence, coverage, and underlying environmental and hygienic conditions is needed. Programs should not skip MDAs, and at prevalences >30%, 7 or more annual MDAs may be required to achieve the target. There are five years left before the 2020 deadline to eliminate blinding trachoma. Low endemic settings are poised to succeed in their elimination goals. However, newly-identified high prevalence districts warrant immediate inclusion in the global program. Intensified application of the SAFE strategy is needed in order to guarantee blinding trachoma elimination by 2020.

## Author Summary

Trachoma, the world’s leading infectious cause of blindness, is scheduled for elimination by 2020. Reaching this elimination target depends on successful implementation of the SAFE strategy (Surgery, Antibiotics, Facial cleanliness, and Environmental improvements). Annual mass antibiotic distributions are key to breaking the cycle of transmission in a community. However, it is not clear how many annual mass treatments need to be carried out in order to achieve elimination. Our study analyzes the effect of mass antibiotic distribution on different baseline prevalence levels of trachoma, in order to assess factors that affect the success of reaching elimination goals. We find that the prevailing belief, which suggests that 3 annual mass treatments can achieve local elimination of trachoma at prevalences between 10–30%, and 5 annual mass treatments for districts above this benchmark, is probably incorrect. In fact, much longer intervals may be required with “business as usual” programmatic strategies, which often include skipped years of treatment. Districts with high prevalence levels may require more intense treatment strategies to eliminate trachoma. Intensified recommendations must be implemented without delay in order to reach the 2020 elimination deadline.

## Introduction

Trachoma remains the world’s leading infectious cause of blindness, although it has disappeared from much of the developed world due to advances in hygiene and sanitation. The World Health Organization (WHO) has classified it amongst the neglected tropical diseases (NTDs), as where it remains, it is concentrated among the world’s poorest populations. These communities live “at the end of the road,” beyond the reach of development infrastructure, and lack access to the basic sanitation measures that prevent disease transmission. Currently, WHO estimates that 232 million people live in endemic areas, 21.4 million have active trachoma, and 7.3 million suffer from trachomatous trichiasis (TT) and are at immediate risk of becoming blind [1–3]. However, through implementation of the SAFE strategy (**S**urgery, **A**ntibiotics, **F**acial cleanliness, and **E**nvironmental improvements), we hope to reduce active disease, defined as trachomatous inflammation—follicular among children aged 1–9 (TF_1–9_) [4] to below 5% prevalence in every endemic district by 2020. As over 100 repeated infections are required to cause the scarring that leads to blindness [5], this will ensure that no one accrues sufficient infections to progress to the disease’s blinding end stages, thus accomplishing elimination of blinding trachoma.

In order to achieve sustainable elimination, effective implementation of each component of the SAFE strategy is essential. Treatment with Zithromax (azithromycin) successfully clears individual infections [6,7], but many factors affect the impact of mass drug administration (MDA) at the population level, such as MDA coverage [8,9] and concurrent implementation of environmental improvements and hygiene education [10,11]. Current recommendations from WHO are to perform at least three annual MDAs prior to an impact survey when baseline TF_1–9_ prevalence is 10–29%, and at least five MDAs before an impact survey when baseline TF_1–9_ prevalence is ≥30% [12]. These benchmarks were instituted in 2010 as an update to the original guidelines from 2006 [13], which proved insufficient for some high endemic areas.

Many perceive these benchmarks to suggest that a certain number of years of treatment “guarantee” elimination, but this may be incorrect. Even in relatively low-endemic regions, elimination may take more than three annual MDAs [14,15]. Three treatment rounds were also not sufficient for sustained elimination at roughly 30% baseline TF_1–9_ prevalence [16]. Modeling suggests that where TF_1–9_ prevalence is ≥50%, five years of annual treatment is likely not enough [17,18]. Indeed, 7–10 MDAs may be necessary [9].

Given the increase in available research and programmatic data, these recommendations can be assessed and refined to allow trachoma control programs to appropriately plan and budget for elimination. In this study, we used a global dataset of baseline and impact surveys to assess the evidence base for the effect of MDA on trachoma prevalence, with the goal of determining whether improved recommendations can be developed in order to improve programmatic efficiency and ensure continuous progress towards elimination.

## Methods

### Database

In order to effectively coordinate the Zithromax donation on behalf of Pfizer, the International Trachoma Initiative (ITI) maintains a comprehensive database of trachoma prevalence and Zithromax treatments performed around the world. This database allows ITI to effectively allocate drugs, and conduct forecasting and planning of programmatic scale-up [19,20]. Data sources include published literature reports and annual applications for Zithromax submitted to ITI, personal communication with national program staff and researchers, and targeted review of other sources. This study includes database updates through February 2014.

Each observation in the database includes the following information, if available: active trachoma prevalence and the clinical sign used as an active indicator (TF or TF/TI), trachomatous trichiasis (TT) prevalence, age range of individuals surveyed for TF and TT, survey location, survey year, survey design and sampling methodology, and data source. Where multiple surveys were conducted at a given location, they were coded to indicate if they preceded or followed treatment. Where treatment was conducted, some entries include estimates of district population, reported antibiotic distribution in doses, and coverage (estimated as doses distributed divided by total population).

There is substantial variation between some of the surveys represented in the database. For example, the indicator used for active trachoma is a measure of circulating disease in a community. Though the WHO standard is to measure trachomatous inflammation—follicular (TF) among children aged 1–9 years (TF_1–9_), some surveys assessed TF among school-aged children or children under 6 years old. All surveys included in the database used the simplified clinical grading system for trachoma [4], but some measured TF as an indicator for active trachoma and others used a combination of TF and TI (trachomatous inflammation, intense).

While cross-sectional population-based prevalence surveys (PBPS) are considered the gold standard for assessing trachoma prevalence at a given location [19,21], data from trachoma rapid assessments (TRAs) and acceptance sampling trachoma rapid assessments (ASTRA) were reported from some locations. The trachoma community experimented over several years with alternative methods for providing evidence to start programmatic implementation, however, neither have been routinely adopted [21]. TRAs are designed to provide biased prevalence estimates, as they prioritize finding trachoma where it exists [22,23]. In most cases, these TRAs were used to determine areas where a PBPS should be implemented. Prevalence surveys are intended to take place using the district as the implementation unit (where district is defined as an administrative unit of 100,000–250,000 people), but are sometimes performed at a larger geographic area, such as the zonal level, if trachoma is expected to be hyperendemic [12]. Sub-district analyses are also required if TF_1–9_ prevalence is below 10% at district level [12].

### Data Cleaning and Abstraction

We assessed the factors affecting change in prevalence over time in pairs of surveys collected at the same location. The database initially contained 2365 surveys. These represented 29 countries and were performed between the years 1992–2013. We censored 156 TRAs and 46 ASTRAs. Of the 2157 remaining surveys, 353 represented follow-up after treatment, 1318 represented baseline that preceded treatment, and the remaining 486 represented surveys that did not prompt treatment. All 1671 surveys that preceded or followed treatment were assigned unique IDs by location and matched. Matches were parsed into pairs corresponding to two prevalence surveys in the same location and ordered chronologically. Matched pairs were merged with data on treatment and coverage that used the same unique IDs by location. In areas where follow-up assessment was conducted at a smaller implementation level than the baseline survey (e.g. district surveys following a zonal survey), the follow-up data was averaged across the original unit of implementation to allow comparison.

We investigated adjustment factors where active disease prevalence was not measured as TF_1–9_. In settings with TF prevalence exceeding 20%, the age-prevalence peak may shift such that younger individuals are more likely to have a greater share of disease burden [5,24–26]. However, data from the PRET trial showed a very high level of correlation between active disease among children 0–5 and 1–9 years old [27,28]. Thus, we did not apply a scaling factor where TF prevalence was assessed among children under six. As the only surveys in the dataset that sampled children aged 6–15 were conducted in Vietnam, where school attendance is high and prevalence peaks among school-aged children [29], no adjustment was applied. If TF/TI was used as an active indicator rather than TF alone, it was adjusted by a factor of 0.87. This was calculated as an average of the relative difference between TF and TF/TI prevalences in published studies [30–33]. Finally, among surveys for which a year range was specified, the survey year was coded as the median of that range or the most recent year of a two-year range.

**Fig 1:**
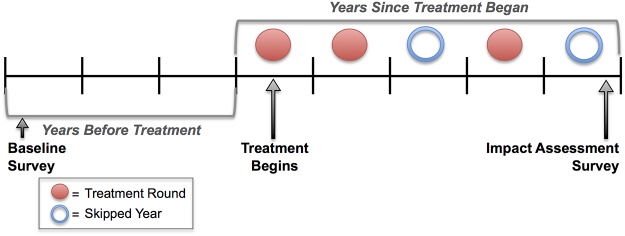
Sample treatment schedule illustrating the variable coding scheme. This represents Years Before (here, 3 years after baseline survey and before MDA starts), Rounds Between (here, 3 MDA rounds between surveys), and Years Since (here, 5 years since treatment began). Skipped years are coded as follows: Skips Between (here, 1 skipped year between treatment rounds), and Total Skips (any years without treatment since treatment began, here, 2).

Pairs were identified as representing MDA if any treatment was recorded between the survey dates, or if ITI coding indicated that MDA had taken place. All other pairs were considered to represent “background” prevalence change. Variables were created representing annual MDAs between treatment (number of MDAs that took place between baseline and follow-up surveys), number of years between surveys, number of years before treatment (years between baseline survey and first MDA), number of years since treatment (years between first MDA and follow-up survey), total annual MDAs (number of MDAs before the follow-up survey, regardless of whether they took place after the baseline survey), skips between (“treatment holiday,” or skipped years between annual MDAs), and total skipped years (any years without treatment before the follow-up survey and after the beginning of treatment). See Fig. 1 for a representation of this coding scheme.

As all temporal information in the database is based on calendar years, discrimination between time intervals smaller than a year was not possible. Thus, a given “year” could be as short as 12 months or as long as 23 (e.g., if a baseline survey took place at the beginning of one calendar year and an MDA took place at the end of the next calendar year). Coding proceeded on the assumption that baseline surveys would be followed by treatment, while impact surveys followed treatment. Instances of anomalous code were manually inspected and cleaned. The final dataset had 170 pairs of surveys corresponding to baseline and follow-up after MDA, and 112 pairs that did not correspond to MDA. All of these represented population-based prevalence surveys.

**Fig 2:**
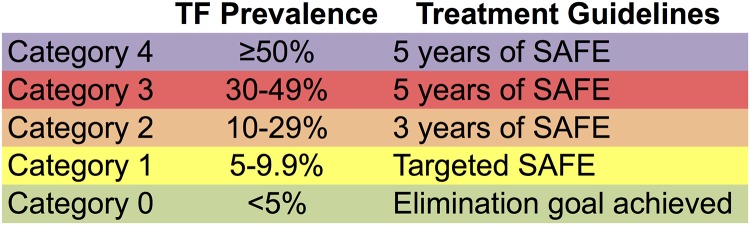
Categories of TF_1–9_ prevalence used in ordinal logistic regression modeling. Categories are based on WHO recommendations for elimination.

In order to perform ordinal logistic regression modeling (described below), we created a categorized ordinal variable for TF_1–9_. TF_1–9_ categories were specified based on the thresholds that define current WHO recommendations for treatment [12]. An additional category, in which prevalence exceeded 50%, was added to represent hyperendemic settings where trachoma is entrenched (see Fig. 2). These thresholds correlate with number of rounds MDA applied, and often years between surveys, and thus categorize the data into similar groups.

Coverage data, applicable only to the treatment dataset (since the background dataset did not by definition involve MDA), was only reported in 2010–2012. Therefore, coverage data was available for the end of the treatment cycle for only those survey pairs whose treatment interval included at least one of these years: this was true of just 52 (approximately 31%) of the survey pairs in the treatment dataset. We therefore omitted this variable from modeling.

### Data Analysis

The final dataset contained 282 pairs of surveys, which were conducted between 1996–2013. We used SAS 9.4 (SAS Institute, Cary, NC, USA) to produce descriptive statistics of the dataset (Table 1). Generalized linear models were fit to the “background” dataset, which represented change in prevalence in the absence of MDA, and the “treatment” dataset, which represented MDA’s effect on prevalence. The outcome variable for each was defined as TF_1–9_ prevalence at follow-up. Stepwise selection and backwards elimination strategies, with entry and stay criteria of α = 0.10, respectively, were used for model building, with all possible variables included at the outset. Aikake Information Criterion (AIC) was used to compare models. The assumption of linearity was confirmed using an overall F test, as well as by plotting the residuals of the explanatory variables. Univariate and multivariate logistic regression models were fitted to banded TF_1–9_ prevalence at follow-up (see Fig. 2 for categories) to demonstrate the odds of reduction to lower categories of follow-up TF_1–9_ prevalence. Stepwise selection and backwards elimination were again used to determine final model candidates. Maximum likelihood was used to estimate the coefficients for model predictors [34]. Collinearity was assessed for linear modeling using variance inflation factors, and for logistic modeling using condition indices and variable decomposition factors, calculated with a SAS macro [35]. Given a condition index of ≥30, we investigated variables associated with decomposition factors ≥0.5 [34].

**Table 1:** Characteristics of survey data, matched on location, from the ITI global prevalence database.

	“Treatment” (with MDA)^a^ (n = 170)		“Background” (no MDA)^a^ (n = 112)	
	No.	%	No.	%
**Countries represented**				
Burkina Faso	18	10.8	37	33.0
Ethiopia	25	15.0	10	8.8
Ghana	23	13.8	0	0.0
Mauritania	20	12.0	0	0.0
Nigeria	4	2.4	50	44.6
Vietnam	25	13.2	0	0.0
Burundi, Guinea, Guinea Bissau, Kenya, Malawi, Mali, Morocco, Mozambique, Nepal, Niger, Sudan, Tanzania, The Gambia	55	32.4	15	13.4
**Baseline TF Prevalence**				
Category 0: <5%	14	8.2	14	12.5
Category 1: 5–9.9%	27	15.9	22	19.6
Category 2: 10–29.9%	96	56.5	48	42.9
Category 3: 30–49.9%	23	13.5	26	23.2
Category 4: >50%	10	5.9	2	1.8
**Follow-up TF Prevalence**				
Category 0: <5%	89	52.4	46	41.1
Category 1: 5–9.9%	30	17.7	21	18.8
Category 2: 10–29.9%	37	21.8	36	32.1
Category 3: 30–49.9%	14	8.2	8	7.1
Category 4: >50%	—	—	1	0.9
**Years Between Surveys**				
1–2 years	25	14.7	1	0.9
3–4 years	51	30.0	13	11.6
5–6 years	43	25.3	36	32.1
7–9 years	35	20.6	14	12.5
>10 years	16	9.4	48	42.9
**Rounds Between Surveys**				
0 Rounds	—	—	112	100
1–3 Rounds	91	53.5	—	—
4–5 Rounds	36	21.2	—	—
>5 Rounds	18	10.6	—	—
* Missing* ^*b*^	*25*	*14*.*7*		
**Years Before Start of Treatment** ^**c**^				
0 Years	56	32.9	—	—
1–2 Years	58	34.1	—	—
3–6 Years	26	15.3	—	—
7+ Years	5	2.9		
* Missing* ^*b*^	*25*	*14*.*7*	—	—
**Years Since Start of Treatment**				
1–3 Years	27	15.9	—	—
4–5 Years	80	47.1	—	—
6+ Years	38	22.4	—	—
* Missing* ^*b*^	*25*	*14*.*7*	—	—
**Coverage Data**				
Any data, 2010–2012	52	28.4	—	—
* Missing*	*131*	*71*.*6*	—	—

^a^Pairs were sorted into the "treatment" dataset if any MDA had occurred in the interval between them.

^b^41 pairs in the treatment dataset were missing data on when treatment occurred.

^c^These values represent the time interval between the baseline survey and the actual start of treatment.

In the treatment dataset, 28 observations coded as representing MDA but missing data on treatment were dropped from the linear and logistic models due to missing predictor values. Pairs dropped included data from Ghana, Nigeria, Tanzania, The Gambia, and Vietnam.

## Results

Using several selection strategies in generalized linear modeling, we included the following variables in the final model for the treatment dataset: baseline TF_1–9_ prevalence (0.13, 95% CI: -0.17, 0.43), rounds of MDA (-2.59, 95% CI: -4.47, -0.71), years since treatment began (1.80, 95% CI: 0.67, 2.93), years before treatment began (-0.94, 95% CI: -1.79, -0.17), and the interaction between rounds of MDA and baseline TF_1–9_ prevalence (0.062, 95% CI: 0.003, 0.12). These were significant at the 0.05 level, with the exception of baseline prevalence, which also exhibited collinearity with the interaction term but had to be retained for a hierarchically well-formulated model. The final multivariate model, specified below, had an r^2^ value of 0.40:

***TFPr2*** = 3.22 + 0.13 * ***TFPr1***–2.59 * ***Rounds MDA*** + 1.80 * ***Years Since Treatment Start*** - 0.94 * ***Years Before Treatment*** + 0.062 * (***TFPr1*** * ***Rounds MDA***)

In contrast, the best model fit to the background dataset (without MDA) accounted for only about 8% of the variation in the data, demonstrating that these model parameters do not do a good job of accounting for TF_1–9_ prevalence change in the absence of treatment.

Univariate ordinal logistic regression performed on the treatment dataset (Table 2) demonstrated that increased baseline TF_1–9_ prevalence was significantly associated with reduced likelihood of achieving lower categories of follow-up TF_1–9_ prevalence. Years since treatment began and total skipped years since treatment began were also significant. Increased number of annual MDAs and years skipped between annual MDAs also showed a non-significant trend towards association with reduced likelihood of reduction.

**Table 2:** Univariate ordinal regression analysis demonstrating the likelihood of a decrease in TF_1–9_ prevalence at follow-up given an increase in continuous predictors.

	“Treatment” (with MDA)^a^ (n = 170)			
	No. (%)	OR	95% CI	*p*-value
Baseline TF Prevalence	**170 (100%)**	**0.93**	**(0.91,0.95)**	**<0.0001**
Years Between Surveys	170 (100%)	1.02	(0.94,1.11)	0.635
Years Before Treatment Start	145 (85%)	1.13	(0.96, 1.32)	0.142
**Years Since Treatment Began**	**145 (85%)**	**0.78**	**(0.66, 0.91)**	**0.002**
Rounds Between Surveys	145 (85%)	0.91	(0.76,1.10)	0.343
Years Skipped During Treatment Interval	149 (88%)	0.81	(0.64,1.03)	0.0850
**Total Skipped Years Since Treatment Began**	**149 (88%)**	**0.83**	**(0.69,1.00)**	**0.050**

^a^TF_1–9_ prevalence at follow-up is measured in five ordered categories (<5%, 5–9.9%, 10–29.9%, 30–39.9%, 40–49.9%, and >50%).

**Fig 3:**
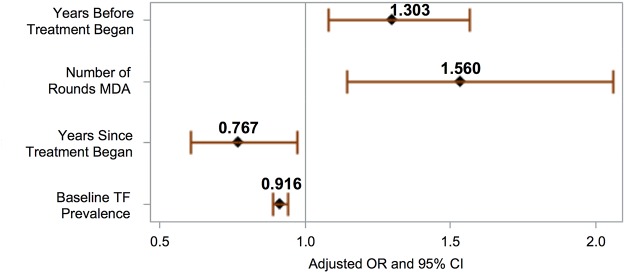
Factors associated with TF_1–9_ prevalence at follow-up in multivariate ordinal logistic regression performed on the treatment dataset (n = 170).

A multivariate ordinal regression model fitted to the treatment dataset was used to model the odds of reduction to a lower category of follow-up TF_1–9_ prevalence. The proportional odds assumption was satisfied for this model. An increase in the following was associated with significantly lower odds of TF_1–9_ prevalence reduction (see Fig. 3): increased baseline TF_1–9_ prevalence (OR = 0.92, 95% CI 0.89–0.94), and years since treatment began (0.77, 95% CI = 0.61–0.97). However, an increase in annual MDAs (OR 1.56, 95% CI 1.16, 2.10) and years before treatment (OR 1.30, 95% CI = 1.08, 1.57) were associated with significantly increased odds of TF_1–9_ prevalence reduction. Censoring of the “super-district” observations, which used mean follow-up TF_1–9_ prevalence to account for baselines measured at the zonal level, did not have a significant effect on these ORs.

**Fig 4:**
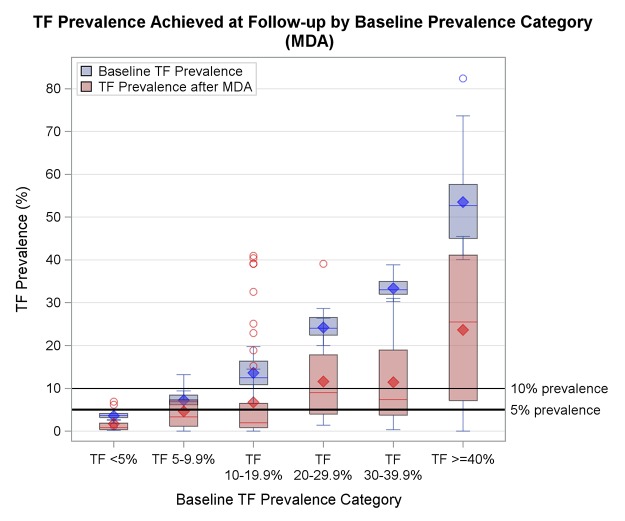
Baseline (blue) and follow-up (red) TF_1–9_ prevalence for survey pairs in the treatment dataset (n = 170), by baseline prevalence category. Data is unmodeled.

**Fig 5:**
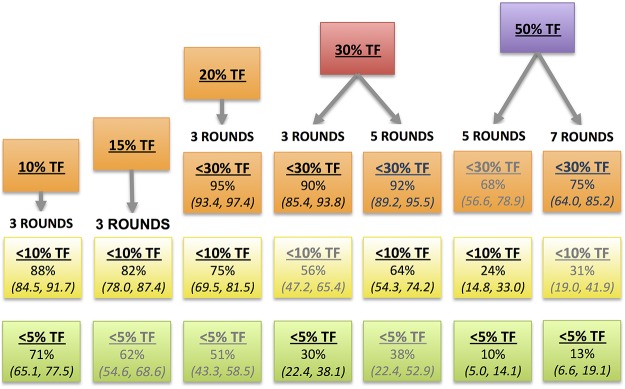
Predicted probabilities (± standard errors) of reduction from baseline TF_1–9_ prevalence to lower prevalence categories. Each probability is cumulative over the lower ordered values, such that the probability of being <30% includes the probability of being <10% and <5%. Predictions not significant at α = 0.10 are in gray.

The unmodeled data demonstrated a general trend of reduction from baseline to follow-up in the treatment dataset, though this was more pronounced at lower baseline prevalence levels (see Fig. 4). Correspondingly, there was a significantly greater probability of reduction to a lower prevalence category at lower TF_1–9_ prevalence levels in the multivariate logistic model (see Fig. 5). While the model predicted a 75% probability of reduction to below 10% given 3 treatment annual MDAs at 20% baseline TF_1–9_ prevalence, the probability of reduction to below 10% given a 30% baseline TF_1–9_ prevalence was 56%. At higher baseline endemicities, the point estimate for probabilities became lower, and the error increased. So while a 56% probability of reduction was predicted for a baseline TF_1–9_ prevalence of 30% given 3 annual MDAs, this was not statistically significant. As number of MDAs increased, the confidence interval narrowed, such that a 64% chance of reduction from 30% baseline was predicted for 5 treatment rounds. Even if the number of MDAs was increased to 10 for an area at 50% endemicity, the probability of reduction (estimated at 42%) was non-significant.

Although various simple measures of skipped years were not significant in the multivariate model, an increase in years since treatment began was significantly associated with reduced odds of prevalence reduction, such that adding a year to the treatment cycle (without a corresponding increase in treatment rounds) led to about a 5% reduction in the probability of success achieving reduction below 10%. The model also predicts increasing success with a waiting period before implementing treatment.

## Discussion

In this study, using data collected in a programmatic context over ITI’s 15-year history, we have demonstrated that the context in which mass drug administration for trachoma is conducted may be as important as the number of annual rounds implemented. Hyperendemic districts (baseline TF_1–9_ prevalence >50%) should implement at least seven MDAs before considering an impact survey, while relatively low-endemic districts (<20% baseline TF_1–9_ prevalence) likely could resurvey after three annual MDAs. However, our models are built using data that represents the imperfect world in which trachoma control programs have operated, with skipped treatment years and little data on antibiotic coverage and improvements in hygiene and sanitation. The context in which MDA is implemented is also crucial, and is likely key to successful elimination of trachoma.

Some of the principles demonstrated by our models regarding treatment context are well recognized. Trachoma tends to decline slowly on its own, probably due to the effects of gradual development and improvements in hygiene and sanitation [36,37]. This is likely represented by the variable for years before treatment, which predicts that in the absence of treatment (or before treatment), there is a modest decrease in prevalence at follow-up. Furthermore, trachoma is more likely to reemerge after treatment in higher prevalence settings [8,18,38,39], while in lower prevalence settings it disappears after treatment [40,41]. The variable for baseline TF_1–9_ prevalence demonstrates that the effect of MDA varies at different endemicities. We had limited ability to investigate interactions between variables due to insufficient power and a small number of potential variables. As such, although the interaction term in the linear models shows that a higher baseline prevalence is less responsive to treatment, neither this term nor a potentially interesting interaction between baseline prevalence and skipped years could be included in the logistic models due to unacceptable levels of multicollinearity. However, in all the models, skipped years, or additional years since treatment began made reduction less likely. We see this effect despite the fact that a single “year” in our data may represent anywhere from 12 to 23 months, given that reporting is agnostic to timing of surveys and treatment during the calendar year.

We assessed the combined effects of these variables by generating predictions for various treatment schemes. The multivariate logistic model predicts that increasing the number of annual MDAs leads to a higher probability of TF_1–9_ prevalence reduction. No matter how many continuous MDAs are conducted, achievement of the elimination target levels becomes less likely as baseline prevalence increases. Of the ten districts in the treatment dataset with baseline TF_1–9_ prevalence >50%, none showed reduction to below 5%, and only one achieved reduction to below 10%, despite the application of up to seven annual MDAs (see Fig. 4). This limits the capacity of the model to predict successful reduction in hyperendemic conditions. Even at TF_1–9_ prevalences between 30–50%, only about half of the districts achieved reduction below 10%.

The model suggests, therefore, that low endemic districts (<20%) are likely to achieve reduction to below 10% after three annual MDAs, and should be resurveyed at that time. However, at 30% baseline TF_1–9_ prevalence, the model predicts a 56% chance of reduction to below 10%. This probability dwindles as baseline TF_1–9_ prevalence increases. From the limited available evidence, even 7 annual MDAs were insufficient in hyperendemic districts (>50% TF_1–9_ prevalence) to make a meaningful public health difference. In such programmatic contexts, over 7 annual MDAs may be necessary to achieve the target. These findings are supported by other studies: in a programmatic context in Mali, three annual rounds of MDA were not sufficient at baseline prevalences of close to 30% [16], while seven to ten years of annual treatment were also suggested by a research study in a hyperendemic setting in Tanzania [9].

Once again, our models do not represent the effect of MDA conducted in controlled conditions. It is likely that many of the districts in our dataset did not achieve their prevalence reduction goals due to inconsistent application of the SAFE strategy. For example, most of the high endemic districts experienced discontinuous treatment. As described, skipped treatment years significantly decrease the probability of TF_1–9_ prevalence reduction. Our models also omit data on other factors known to influence the effect of MDA, such as treatment coverage [8]. Coverage data was available in such a small subset of surveys that it could not be included in our models; less than half of the districts surveyed in 2010–12 reported any kind of MDA coverage measures to ITI. However, even if more programs provided these estimates, the quality of coverage data currently collected by trachoma control programs is known to vary greatly [42].

We also lack measures of hygiene and environmental factors, the F and E components of the SAFE strategy. Reduction in trachoma has been associated with clean faces and hygiene indicators [43], latrine provision [24,44], and insecticide spraying to control flies, which can act as trachoma vectors where they are prevalent [45,46]. Direct causative evidence is lacking to guide the development of metrics that could be used by control programs. Nonetheless, the endemic equilibrium that leads to reemergence of trachoma is likely dependent on environmental factors [5,17,39]. If the setting in which antibiotic treatment is applied is unchanged, “elimination” will be transient at best.

Despite these omissions, our results are valuable precisely because they represent the effect of MDA as it is conducted by trachoma control programs. Although low endemic districts are likely to succeed in their elimination goals under the current WHO recommendations, we must consider carefully how to support the remaining districts with baseline TF_1–9_ prevalence over 30%. With just under five years left before the 2020 elimination goal, those districts must plan for intensified treatment programs. They may consider alternatives such as targeted treatment [47] or biannual treatment [8,48]. There may be substantial cost savings associated with proposed integration of efforts to survey and distribute treatment with programs for other NTDs [49–51]. Most importantly, we must recognize that in the imperfect context in which programs on the ground operate, adding more annual MDAs without regard to coverage, programmatic continuity, and underlying environmental context will not guarantee trachoma elimination.

In order to continue our progress towards trachoma elimination, we must emphasize the WHO recommendations that call for programmatic continuity, which should be attainable even in countries where program implementation is difficult, given increased donor support. We must also emphasize the importance of antibiotic coverage, hygiene education, and sanitation improvements. This should start at the level of the data we collect. We cannot track progress, measure success, or even understand what success looks like for variables we do not measure.

Trachoma serves as an object lesson that antibiotic interventions, such as azithromycin mass treatment, can only go so far in the context of poor development. With increasing rounds of MDA, we may eventually reduce TF_1–9_ prevalence to below 5%, even in the most high-endemic districts remaining. Our data suggests that such districts ought to prepare for extended MDA timelines. However, we should not rely on antibiotics alone to achieve trachoma elimination. The most effective and efficient solution is likely to implement all aspects of the SAFE strategy, which recognizes that though high-coverage, continuous MDAs are essential, clean water and good hygiene may be as important. For programs seeking real and sustainable elimination, it may be that no amount of time is long enough to achieve trachoma elimination without lasting change of the environment in which it persists.
